# Identification of ACTA2 as a Key Contributor to Venous Malformation

**DOI:** 10.3389/fcell.2021.755409

**Published:** 2021-11-08

**Authors:** Song Wang, Zifu Zhou, Jing Li, Yu Wang, Hongwen Li, Renrong Lv, Guangqi Xu, Jian Zhang, Jianhai Bi, Ran Huo

**Affiliations:** ^1^Department of Burn and Plastic Surgery, Shandong Provincial Hospital, Cheeloo College of Medicine, Shandong University, Jinan, China; ^2^Department of Burn and Plastic Surgery, Shandong Provincial Hospital Affiliated to Shandong First Medical University, Jinan, China

**Keywords:** ACTA2, proteomics, zebrafish, vascular development, venous malformation (VM)

## Abstract

**Objectives:** Proteomics and high connotation functional gene screening (HCS) were used to screen key functional genes that play important roles in the pathogenesis of venous malformation. Furthermore, this study was conducted to analyze and explore their possible functions, establish a gene mutation zebrafish model, and perform a preliminary study to explore their possible pathogenic mechanisms in venous malformation.

**Methods:** Pathological and normal tissues from patients with disseminated venous malformation were selected for Tandem Mass Tag (TMT) proteomics analysis to identify proteins that were differentially expressed. Based on bioinformatics analysis, 20 proteins with significant differential expression were selected for HCS to find key driver genes and characterize the expression of these genes in patients with venous malformations. *In vitro* experiments were then performed using human microvascular endothelial cells (HMEC-1). A gene mutant zebrafish model was also constructed for *in vivo* experiments to explore gene functions and pathogenic mechanisms.

**Results:** The TMT results showed a total of 71 proteins that were differentially expressed as required, with five of them upregulated and 66 downregulated. Based on bioinformatics and proteomics results, five highly expressed genes and 15 poorly expressed genes were selected for functional screening by RNAi technology. HCS screening identified ACTA2 as the driver gene. Quantitative polymerase chain reaction (qPCR) and western blot were used to detect the expression of ACTA2 in the pathological tissues of patients with venous malformations and in control tissues, and the experimental results showed a significantly lower expression of ACTA2 in venous malformation tissues (*P* < 0.05). Cell assays on the human microvascular endothelial cells (HMEC-1) model showed that cell proliferation, migration, invasion, and angiogenic ability were all significantly increased in the ACTA2 over-expression group (*P* < 0.05), and that overexpression of ACTA2 could improve the inhibitory effect on vascular endothelial cell proliferation. We constructed an ACTA2-knockdown zebrafish model and found that the knockdown of ACTA2 resulted in defective vascular development, disruption of vascular integrity, and malformation of micro vein development in zebrafish. Further qPCR assays revealed that the knockdown of ACTA2 inhibited the Dll4/notch1 signaling pathway, Ephrin-B2 signaling pathway, and vascular integrity-related molecules and activated the Hedgehog signaling pathway.

**Conclusion:** This study revealed that ACTA2 deficiency is an important factor in the pathogenesis of venous malformation, resulting in the disruption of vascular integrity and malformed vascular development. ACTA2 can be used as a potential biomarker for the treatment and prognosis of venous malformations.

## Introduction

Venous malformation (VM) is a common congenital vascular malformations comprising dilated and tortuous veins of variable size with a sparse and disorganized arrangement of vascular smooth muscle cells, which gradually expand and tortuously form clusters with growth and development (Dompmartin et al., 2010; Wassef et al., 2015). It can occur in all parts of the body, with the oral and maxillofacial areas, head and neck, and extremities being the main sites of onset (Judith et al., 2014). The main clinical manifestations are swelling, pain, bleeding, and limited range of motion, which eventually form irreversible dysfunction, leading to severe dysfunction or severe bleeding and becoming life-threatening when the lesions involve vital organs (Dompmartin et al., 2010; Judith et al., 2014; Manoli et al., 2015).

Several pathogenic mutations associated with venous malformation have been recently identified, and attempts have been made to elucidate the pathophysiological pathways involved in their development. Studies have shown that somatic mutations in genes, such as TIE2 (TEK), PIK3CA, and MAP3K3, are associated with VM (Couto et al., 2015; Natynki et al., 2015; Castillo et al., 2016; Seront et al., 2018). Based on the mode of inheritance, VM can be divided into disseminated VM and familial venous malformations (VMCM), of which the familial type is more complex and difficult to treat. Several studies have found that many VMCM patients have specific genetic mutations, namely TIE2-R849W (Calvert et al., 1999; Shu et al., 2012).

In this study, Tandem Mass Tag (TMT) was used to screen proteins that were differentially expressed between patients with disseminated venous malformations and healthy people. The differentially expressed proteins were analyzed by bioinformatics, and their cellular functions and enriched classical molecular pathways were characterized. Twenty differentially expressed proteins were selected for further functional screenings by high-content screening (HCS) at the gene level. The differentially expressed gene ACTA2 was identified and validated in the tissues of patients with venous malformations by RNA and protein expression profiles. Cell assays were performed using human microvascular endothelial cells (HMEC-1) to explore the function of the ACTA2 gene. Finally, we established the ACTA2 zebrafish model to observe how mutations affect the development of the caudal venous plexus and to investigate the related mechanism.

## Materials and Methods

### Sample Collection

Ten patients with venous malformations from 2019 to 2020 in the Department of Plastic Surgery of Shandong Provincial Hospital were selected, and surgically excised venous malformation tissues were collected (Table 1). Ten cases of normal tissues were selected as the control group. The tissue specimens were transferred to liquid nitrogen for preservation. Of these samples, three pairs were used for quantitative proteomics analysis, while all samples were assessed for further validation. Postoperative pathological diagnoses were made by two independent pathologists. The study was approved by the Ethics Committee of Shandong University Provincial Hospital.

**TABLE 1:** Clinical characteristics of patients.

ID	Age	Gender	Location	Pathological classification
1	16	Male	Chest	VM
2	9	Female	Leg	VM
3	12	Female	Back	VM
4	12	Male	Leg	VM
5	25	Female	Foot	VM
6	13	Male	Perineum	VM
7	6	Male	Leg	VM
8	10	Female	Face	VM
9	37	Female	Thigh	VM
10	1	Male	Wrist	VM

### Quantitative Proteomics Analysis by Tandem Mass Tag

#### Protein Extraction, Quantification, and Proteolysis

Each of the control and experimental groups contained three tissue samples that were labeled as A1, A2, and A3 and B1, B2, and B3, respectively. An appropriate amount of SDS Lysis Buffer was added to the samples and transferred to the Lysing Matrix A tube for homogenization with MP homogenizer (24.2, 6.0 M/S, 60 s, twice). After sonication (100 W, 10 s, 10 times), samples were heated in a boiling water bath for 10 min. After centrifugation at 14000 × *g* for 15 min, the supernatant was filtered using a 0.22 μm centrifuge tube, and the filtrate was collected. Protein quantification was performed by the BCA method. The samples were aliquoted and stored at −20°C. Then, 100–200 μg of each sample was retrieved and underwent proteolysis by the filter-aided sample preparation method (Nasevicius and Ekker, 2000), and peptides were quantified by measuring the OD_280_ on the Nanodrop.

#### Tandem Mass Tag Labeling and High pH RP Peptide Grading

Firstly, 100 μg of the peptide was taken from each sample and labeled by the TMT labeling kit (Thermo, United States) following the manufacturer’s instructions. Then, 100 μL of the sample was placed in a centrifuge tube, mixed with TMT solution, vortexed and centrifuged, and left at room temperature for 1 h. The labeled samples were mixed, vortexed and centrifuged, vacuum freeze-dried, and stored at −20°C. The labeled peptides from each group were mixed and graded using the Agilent 1260 Infinity II HPLC system (Agilent, Germany).

#### Mass Spectrometry Analysis and Identification

Samples were separated using the nanoliter flow rate EASY-nLC System (Thermo, United States). Samples were loaded by autosampler onto an analytical column (Thermo Fisher Scientific Acclaim PepMap RSLC 50 μm × 15 cm, nano viper, P/N164943) for detection and analysis. After separation by chromatography, samples were analyzed by mass spectrometry using a Q Exactive^TM^ plus mass spectrometer (Thermo, United States). The raw data for mass spectrometry analysis were retrieved as raw files, and the software Mascot 2.6 and Proteome Discoverer 2.15 (Thermo, United States) were used for library identification and quantitative analysis.

### Bioinformatics Analysis

Gene Ontology (GO) analysis was performed on the collection of differentially expressed proteins, including biological process (BP), molecular function (MF), and cellular component (CC), to explore the function of the target proteins. The kyoto encyclopedia of genes and genomes (KEGG) Orthology (KO) and Links Annotation were used for the pathway enrichment analysis. The target protein sequences were first KO categorized by comparing to the KEGG GENES database, and information on the pathways involved in the target protein sequences was automatically obtained according to the KO categorization.

### High-Content Screening Cell Proliferation Assay

The differentially expressed genes were selected based on the results of proteomics and bioinformatics analysis. Multi-target RNAi lentiviral vectors (mix) with a green fluorescent protein (GFP) were prepared for transfection of HMEC-1, and the corresponding control vectors were also prepared. Two-to-three days after the HMEC-1 were transfected with lentiviral vectors, they were inoculated into 96-well plates, and their GFP expression was checked by fluorescence microscopy. When cell fusion reached 70–90%, cells were collected for further experiments. Cell growth was monitored with the Celigo (Nexcelom) high-throughput screening system, which is based on fully automated image acquisition and image data analysis, and light emission was collected from fluorescence-stimulated targets. The targets were the virus-infected GFP-expressing cells, which grew in the 96-well plates. Celigo identified the cells with green fluorescence and took pictures. The images were then analyzed by software to calculate the number of cells in different groups in the plate. After five consecutive days, a cell growth curve was plotted, and statistical analysis was performed on cell proliferation. On day 5, the fold-change values were calculated by comparing the proliferation rate of each group to that of the negative control (NC) group (fold-change of counted cells in the proliferation test NC group/fold-change of counted cells in the experimental group).

### Quantitative Real-Time Polymerase Chain Reaction (RT-PCR)

Tissue samples were subjected to total RNA extraction (Trizol kit from Shanghai Pufei). Samples were received according to kit instructions and reverse transcribed to obtain cDNA (Promega M-MLV kit). The reaction system was configured for Real-Time Polymerase Chain Reaction (RT-PCR) in two steps and a melting curve was plotted. Fluorescent amplification was performed using SY and LCPCR amplifiers. Relative quantitative analysis F = 2- Δ Δ Ct, Δ Ct = target gene Ct – internal reference gene Ct; - Δ Δ Δ Ct = mean Δ Ct of NC group – Δ Ct of each sample; 2- Δ Δ Δ Ct reflected the relative expression level of each sample relative to the target gene of samples in the NC group.

### Western Blot

Total protein was extracted from the tissues using RIPA lysate (Dingguo Biotech, China). Protein concentration was determined using Bicinchoninic Acid (BCA) Protein Assay Kit (Protein Biotechnology, China). 10% SDS-PAGE electrophoresis was performed. After electrophoresis, proteins were transferred to PVDF (Immobilon-P, Cat. No. IPVH00010) using a transfer electrophoresis device with 150 min of electrotransfer at 4°C and 300 mA constant current. Antibody hybridization: PVDF membranes were closed with a blocking buffer (TBST solution containing 5% skimmed milk) at room temperature for 1 h. The blocking buffer was diluted with primary antibody and then incubated with the closed PVDF membranes at room temperature overnight at 4°C, and the membranes were washed with TBST four times for 8 min each. The PVDF membrane was incubated for 1.5 h at room temperature with the corresponding secondary antibody diluted in the blocking buffer and washed with TBST four times for 8 min each. The protein signal was detected using the ECL kit (Millipore, United States).

### Cell Culture and Transfection

The HMEC-1 cell line (CRL-3243) was purchased from the ATCC cell bank. HMEC-1 cell lyophilization tubes were removed from liquid nitrogen and quickly placed in a 37°C water bath and shaken. After complete thawing, the tubes were centrifuged briefly, sterilized with a 75% alcohol wipe, and transferred to a biosafety cabinet. The supernatant was discarded, and the cells were resuspended and inoculated using a complete medium, gently shaken, and placed in a 5% CO_2_ incubator at 37°C. Passaging was performed when the cells reached approximately 80% confluence to maintain good cell growth. HMEC-1 in the logarithmic growth phase was trypsin digested, and 5 × 10^4^ cells/mL cell suspension was made using a complete medium. About 1 mL of cell suspension was inoculated into 12-well culture plates, and the culture was continued to ensure that the spread reached approximately 20% at the time of infection. A 500 uL of infection solution was replaced, and 2.86 uL of virus (7.0E + 8 TU/mL) was added for infection. After 16 h of infection, the fresh culture medium was replaced, and the culture was continued at 37°C. After 72h of infection, the infection efficiency was observed under a fluorescent microscope and photographed. Cells with good growth and successful infection were used for subsequent cytological tests.

### Oris^TM^ Cell Migration Assay

The Oris^TM^ stoppers were sterilized in alcohol and placed in a 96-well plate. Then, 3–5 × 10^4^ infected cells were seeded into the wells according to the experimental design groups so that the cells could reach 90% confluence the following day. The next day, cells were gently rinsed with PBS 2–3 times, and 1% FBS medium was added to the cell culture. The plate was incubated in a 37°C, 5% CO_2_ incubator. Images were captured by Celigo at the appropriate time points three times (generally 0, 8, 16, 24 h, etc.). By adjusting the parameters of the analysis settings, the area of the cells with white or green fluorescence in each scanned well was accurately calculated. Based on the cell area values and time points, differences in the migration ability of tumor cells were determined.

### Cell Invasion Assay

The kit (Corning, Cat. No. 354480) was removed, and the chambers were placed in a new 24-well plate with 500 μL of the serum-free medium in each of the upper and lower chambers and placed in an incubator at 37°C for 2 h to allow rehydration of the Matrigel (Corning, Cat. No. 356234) stromal layer. Serum-free HMEC-1 suspension was prepared and counted for 105/well (24-well plate). After rehydration of the Matrigel matrix, all chambers were transferred to new well plates, the medium was removed from the upper chamber, 200 μL of cell suspension was added, and 750 μL of 30% FBS medium was added to the lower chamber. They were incubated for 24 h in a 37°C incubator. The medium was removed, and the non-invasive cells were gently removed from the small chamber with a cotton swab. After staining the transferred cells with 2–3 drops of staining solution (Sigma, Cat. No. 32884) onto the lower surface of the membrane for 3–5 min, the chambers were soaked and rinsed several times and air-dried. Microscopic photographs were taken and counted at 200X for data analysis to compare the difference in the invasion ability of the cells between the experimental and control groups.

### Cell Cycle Assay

Cells were grown to 80% confluence in 6 cm diameter dishes. After cells were trypsinized and resuspended into cell suspensions, they were collected in 5 mL centrifuge tubes with three replicate wells per group. After 1300 rpm centrifugation for 5 min, the supernatant was discarded, and cells were washed once with pre-chilled D-Hanks (pH 7.2–7.4) at 4°C. Cells were pelleted by centrifugation at 1300 rpm for 5 min and fixed by pre-chilled 75% ethanol at 4°C for 1 h. After cells were washed again with D-Hanks, they were centrifuged at the same speed for the same period. The cell staining solution was prepared as follows: 40 × PI stock solution:100 × RNase stock:1 × D-Hanks = 25:10:1000. Depending on the number of cells, 0.6–1 mL of cell staining solution was added to resuspend the cells so that the cell passage rate was 300–800 cells/s when tested on the instrument.

### Angioplasty Experiments

After HMEC-1 cells were infected, 2 × 10^5^ cells were spread in 6-well plates, walled, washed twice with DPBS (Corning, Cat. No.21-031-CVR), changed to serum-free medium, and cultured for 24 h. The supernatant was collected. Matrigel was removed from −20°C 1 day in advance and left overnight at 4°C. After Matrigel was sufficiently melted, Matrigel was spread in pre-chilled 96-well plates at 70 μL per well and solidified at 37°C for 30 min. HMEC-1 cells were digested, washed 2–3 times with serum-free medium, resuspended to 3 × 10^4^ cells/100 μL with cell culture supernatant, and spread in 96-well plates. After incubation at 37°C for a preset time (2–4 h), the old solution was discarded, and 50 μL of Calcein-AM (Thermo, Cat. No.C3100MP) at a concentration of 0.2 μM was added to each well and incubated at 37°C for 5–10 min. Observation under a fluorescence microscope indicated that the cells had been stained. The plates were placed in a CQ1 instrument and swept to obtain pictures and data.

### Zebrafish Care and Maintenance

Adult wild-type AB strain zebrafish were maintained at 28.5°C on a 14 h light/10 h dark cycle. Five to six pairs of zebrafish were set up for nature mating every time. On average, 200–300 embryos were generated. Embryos were maintained at 28.5°C in fish water (0.2% Instant Ocean Salt in deionized water). The embryos were washed and staged. The establishment and characterization of fli1a-EGFP transgenic lines have been described elsewhere. The zebrafish facility at SMOC (Shanghai Model Organisms Center, Inc.) is accredited by the Association for Assessment and Accreditation of Laboratory Animal Care (AAALAC) International.

### Zebrafish Microinjections and Angiogenesis Studies

Gene Tools, LLC^1^ designed the morpholino (MO). Antisense MO (GeneTools) were microinjected into fertilized one-cell stage embryos according to standard protocols (Nasevicius and Ekker, 2000). The zebrafish ACTA2 gene was targeted by two specific morpholino antisense strategies to prevent either the translation of the zebrafish gene (ATG-MO) or proper splicing of exon2 (E2I2-MO). The sequences of the ACTA2 translation-blocking and splice-blocking morpholinos were 5′- CTTCGT CGTCACACATTTTCAGCTC -3′ (ATG-MO) and 5′- CTTGTGGTACAATAGGTG GTTTACC -3′ (E2I2-MO), respectively. The sequence for the standard control morpholino was 5′- CCTCTTACCTCAGTTACAATTTATA -3′ (Gene Tools). The amount of the MOs used for injection was as follows: Control-MO and E2I2-MO, 4ng per embryo; ATG-MO, 4ng per embryo. Primers spanning acta2 exon 1 (forward primer: 5′- CTCCTTGTTTGGGATGTTAGAG -3′) and exon 3 (reverse primer: 5′- CCTCATCACCAACGTAACTATC -3′) were used for RT-PCR analysis for confirmation of the efficacy of the E2I2-MO. The primer ef1α sequences used as the internal control were 5′- GGAAATTCGAGACCAGCAAATAC -3′ (forward) and 5′- GATACCAGCCTCAAACTCACC -3′ (reverse).

To evaluate blood vessels formation in zebrafish, fertilized one-cell fli1a-EGFP transgenic lines embryos were injected with ACTA2-MO and control-MO. At 50-hpf, embryos were dechorionated, anesthetized with 0.016% MS-222 (tricaine methanesulfonate, Sigma-Aldrich, St. Louis, MO, United States). Zebrafish were then oriented on the lateral side (anterior, left; posterior, right; dorsal, top), and mounted with 3% methylcellulose in a depression slide for observation by fluorescence microscopy. The phenotypes of complete intersegmental vessels (ISVs) (i.e., the number of ISVs that connect the DA to the DLAV), CVP (caudal vein plexus) were quantitatively analyzed.

### Image Acquisition

Embryos and larvae were analyzed with Nikon SMZ 18 Fluorescence microscope and subsequently photographed with digital cameras. A subset of images was adjusted for levels, brightness, contrast, hue, and saturation with Adobe Photoshop 7.0 software (Adobe, San Jose, CA, United States) to optimally visualize the expression patterns. Quantitative image analyses processed using image-based morphometric analysis (NIS-Elements D4.6, Japan) and ImageJ software (U.S. National Institutes of Health, Bethesda, MD, United States^2^. Ten animals for each treatment were quantified and the total signal per animal was averaged.

### Statistical Analysis

All data were presented as the mean ± standard error of mean (SEM). Student’s *t*-test (two-tailed) was used for data analysis. Statistical analysis and graphical representation of the data were performed using GraphPad Prism 5.0 (GraphPad Software, San Diego, CA, United States). *P* < 0.05 was considered to be statistically significant.

## Results

### Quantitative and Statistical Analysis of Proteins Involved in Venous Malformations

**FIGURE 1:**
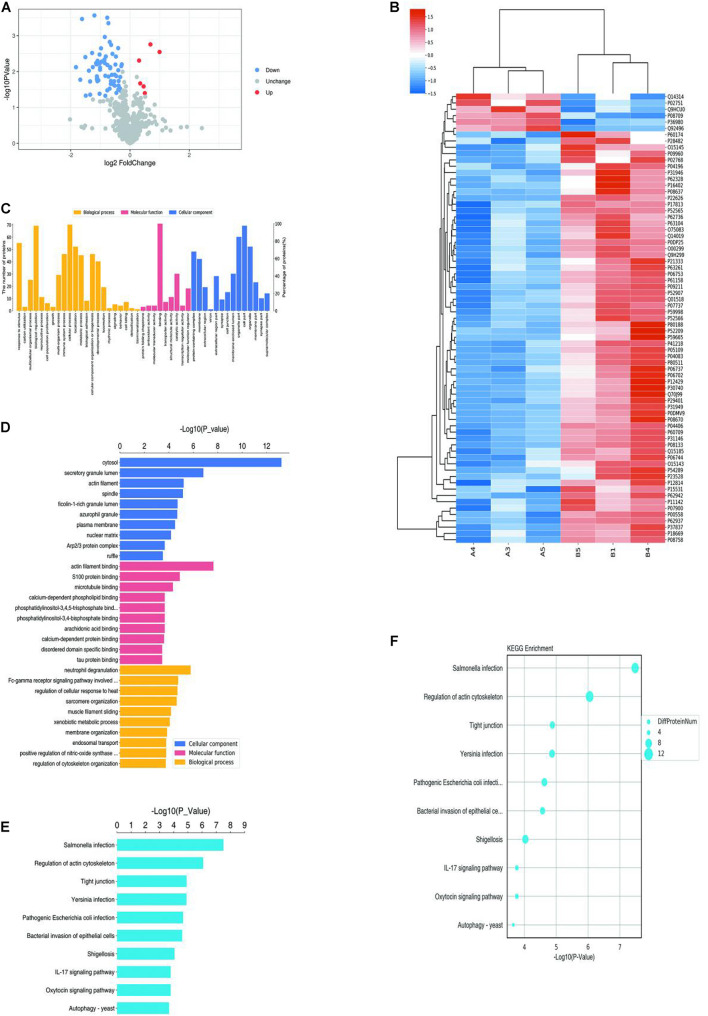
Proteomics and bioinformatics analysis of proteins involved in VM. **(A)** Volcano plots for the differential expression of proteins between VM and the control group (Red—high expression; blue—low expression). **(B)** Heatmap for the differential expression of proteins between VM and control group (Red indicates high expression; blue indicates low expression). **(C)** Gene Ontology (GO) functional annotation analysis of differentially expressed proteins. **(D)** GO functional enrichment analysis of differentially expressed proteins. **(E)** KEGG functional enrichment analysis of differentially expressed proteins. **(F)** KEGG functional enrichment analysis bubble diagram of differentially expressed proteins.

A total of 7408 peptides and 801 protein groups were identified in venous malformations and normal tissues. Proteins with expression differences greater than 1.2-fold (upregulated or downregulated) and a *P* value of less than 0.05 were identified as differentially expressed proteins. As a result, 71 differentially expressed proteins were identified, with five of them being upregulated and 66 being downregulated. Quantification results of the proteins were presented in the form of volcano plots, in which red dots indicate the proteins that were significantly upregulated, blue dots indicate that the proteins were significantly down-regulated, and gray dots indicate proteins with no differential expression (Figure 1A). Clustering analysis was also performed on these proteins, and the results suggested that there was significant differential expression of proteins in venous malformation tissues compared to normal tissues, where red represents upregulated molecules and blue represents down-regulated molecules (Figure 1B).

### Bioinformatic Analysis

Gene ontology (GO) analysis was performed on the differentially expressed proteins, including BP, MF, and CC (Figures 1C,D). In BP analysis, the differentially expressed proteins primarily focused on the biological regulation, cellular process, and regulation of cytoskeleton organization. In MF analysis, differentially expressed proteins primarily focused on actin filament binding, molecular transducer activity, and binding. In CC analysis, differentially expressed proteins primarily focused on the cytosol, organelles, and cell part.

KEGG Automatic Annotation Server software was used to annotate the KEGG pathways of the target protein collection, and the results showed that the KEGG pathways of differentially expressed proteins primarily included Salmonella infection, the IL-17 signaling pathway, and regulation of the actin cytoskeleton (Figures 1E,F).

### High-Content Screening Suggested ACTA2 as a Potential Marker for Venous Malformation

Five highly and 15 lowly expressed protein genes were selected based on bioinformatic analysis and proteomics results for the RNAi functional screening test. The upregulated genes were CFHR2, MMP2, COMP, F7, and CD248. The downregulated genes were MAPK1, HSPA8, HSP90AA1, MMP9, ACTA2, YWHAB, HRG, TFPI, TFPI, ACTG1, ACTG1, GSTP1, GAPDH, GAPDH, and S100A9. Based on the HCS platform, *in vitro* knockdown and overexpression of HMEC-1 models were established for the HCS of venous malformations, and 20 selected genes were tested. Lentivirus was used to construct overexpression and knockdown cell models for the corresponding genes. Plasmids were used to construct overexpression cell models for the five upregulated genes. Three RNAi interference sites were designed for 15 downregulated genes, and three plasmids carrying different target sites were packaged into the lentivirus mix. Cells were transfected and cultured in 96-well plates then counted by Incucyte.

**FIGURE 2:**
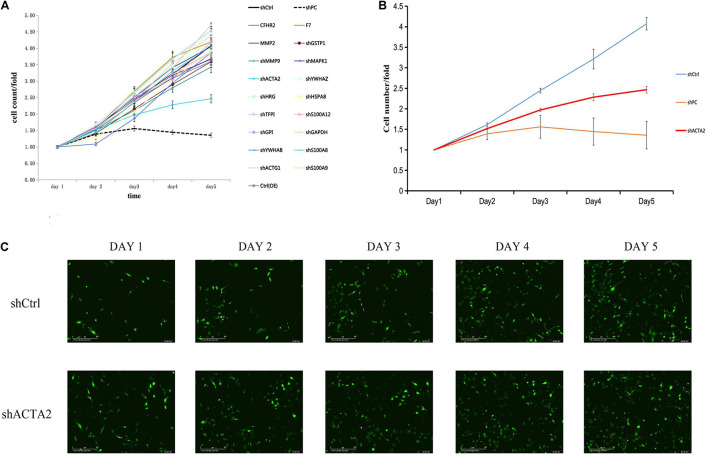
HCS screening identified ACTA2 as a critical gene in promoting VM proliferation. **(A,B)** A total of 20 genes were selected for validation by high-content screening. PC: positive control siRNA targeting b-actin. **(C)** Representative fluorescence images of high-content siRNA screening for ACTA2.

The HCS screening results showed that among the 20 genes to be tested, the shACTA2 group with a fold-change > 1.5 was the differentially expressed gene identified in this experiment (proliferation inhibition-positive cell group) (Figure 2).

The Actin alpha 2 (ACTA2) gene is widely expressed in most cells, and its mutations have been found to cause a variety of vascular diseases, such as thoracic aortic disease, coronary artery disease, stroke, moyamoya disease, and multisystem smooth muscle dysfunction syndrome.

### Lower Expression of ACTA2 in Patients With Venous Malformation

**FIGURE 3:**
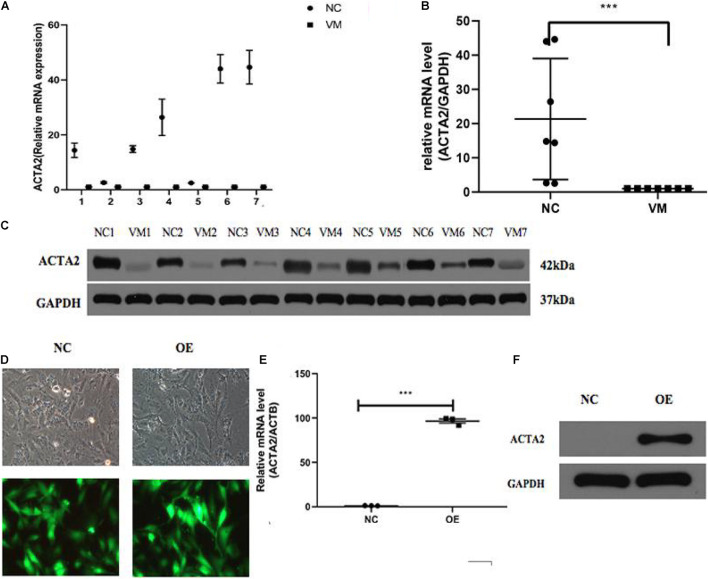
Lower expression of ACTA2 in VM tissues. **(A,B)** The RNA expression level of ACTA2 in 7 pairs of normal and VM tissues. **(C)** Western blotting shows ACTA2 protein expression in 7 pairs of normal and VM tissues. **(D)** Light (up) and fluorescence (down) microscopy images confirming successful transduction of HMEC1 with LV-ACTA2(27000-1) virus and the control group. **(E)** ACTA2 expression levels were assessed by real-time RT-PCR, with GAPDH as an internal control. **(F)** ACTA2 expression levels were assessed by Western blotting. *P < 0.05, **P < 0.01, ***P < 0.0001.

qPCR was used to detect the expression of ACTA2 in pathological tissues of patients with venous malformation and control tissues. The experimental results are shown in Figures 3A,B, in which there was a significantly low expression of ACTA2 in venous malformation tissues relative to that in normal tissues (*P* < 0.05). Western blotting results showed that the protein expression of ACTA2 was lower in venous malformation tissues than in control tissues (Figure 3C).

### Effect of the Overexpression of ACTA2 on the Migration and Invasion Ability of HMEC-1

Considering lower expression of ACTA2 in patients with venous malformation, we performed over-expression of ACTA2 in HMEC1 cells. We constructed LV-ACTA2(27000-1) lentivirus-transfected cells using HMEC1 as the study object, and observed the cells successfully transfected with LV-ACTA2(27000-1) virus overexpressing ACTA2 and negative control virus under a fluorescence microscope after 72 h (Figure 3D). RT-PCR assay showed that the expression of TIE2 was significantly higher in the over-expression (OE) group than in the control group (Figure 3E), and western blot showed significant overexpression of ACTA2 gene in the OE group (Figure 3F). The cells were then used for subsequent functional experiments.

**FIGURE 4:**
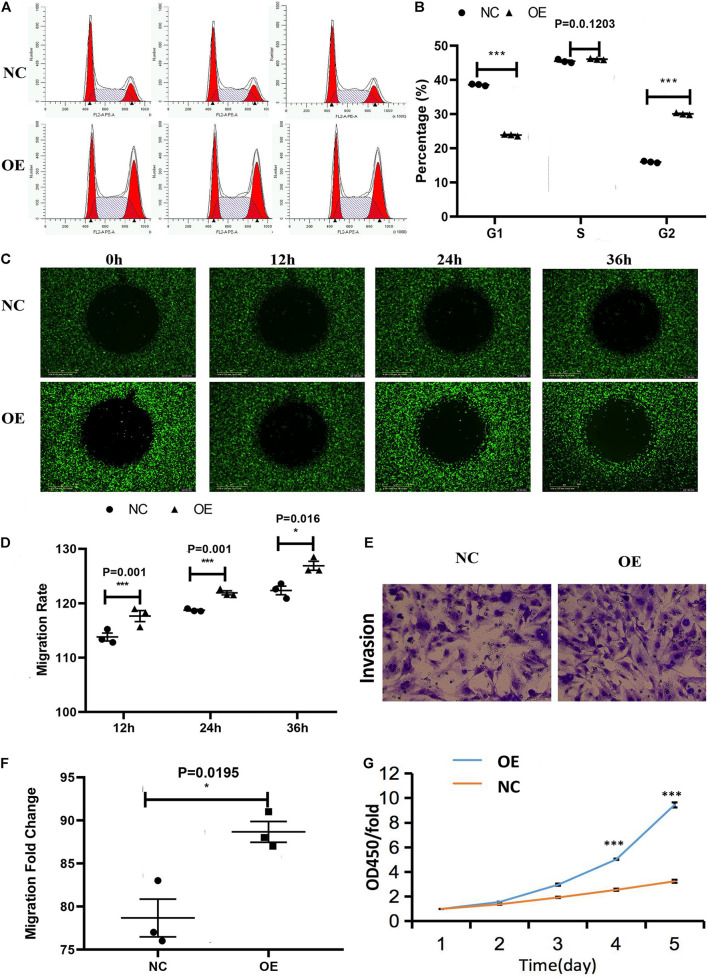
Effect of the overexpression of ACTA2 on the migration and invasion ability of HMEC-1. **(A,B)** Cell cycle distribution was determined by flow cytometry after propidium iodide (PI) staining. **(C,D)** Oris^TM^ plate Wound-healing assay for the migration of HMEC1 after ACTA2 overexpressing. **(E,F)** Invasion assay for the invasion of HMEC1 after ACTA2 overexpressing. **(G)** CCK-8 assay for the proliferation of HMEC1 after ACTA2 overexpressing. *P < 0.05, **P < 0.01, ***P < 0.0001.

The cell cycle assay revealed that the ACTA2 group had increased cells in the G1/G2 phase (*P* < 0.05), and decreased cells in the S phase (*P* < 0.05), while the NC group was just the opposite (Figures 4A,B). The effect of ACTA2 overexpression on vascular endothelial cell migration was detected using Oris^TM^ plate wound-healing, and the results revealed that the migration ability of cells in the overexpression group was higher than that in the control group (*P* < 0.05) (Figures 4C,D). As for the invasion ability of cells, the invasion assay results showed that the invasion ability in the overexpression group was significantly higher than that in the control group (*P* < 0.05) (Figures 4E,F). Moreover, we used the Cell Counting assay (CCK-8) to analyze the effect of ACTA2 on the proliferation of HMEC-1. Overexpression of ACTA2 significantly increased the proliferative ability of HMEC-1 (*P* < 0.05) (Figure 4G). These findings indicate that the overexpression of ACTA2 promotes the proliferation, migration, and invasion ability of human vascular endothelial cells.

### Effect of the Overexpression of ACTA2 on Angiogenesis

**FIGURE 5:**
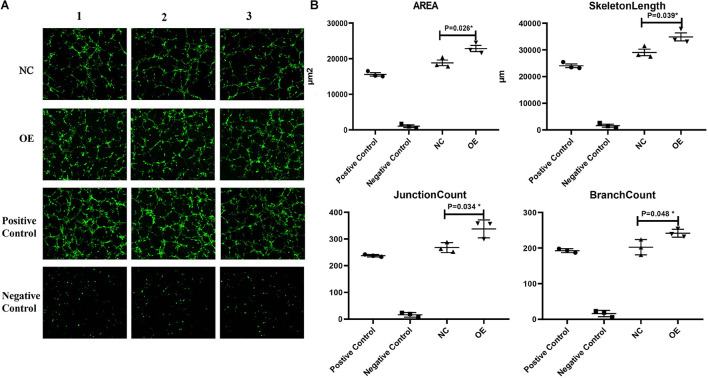
Effect of overexpression of ACTA2 on angiogenesis. **(A)** Representative fluorescence images of angiogenesis in the positive control, negative control, NC, and ACTA2 overexpressing group. **(B)** Quantification of Area, Skeleton Length, Junction Count, Branch Count of positive control, negative control, NC and ACTA2 overexpressing group. *P < 0.05, **P < 0.01, ***P < 0.0001.

The *in vitro* angiogenesis assay was used to test whether ACTA2 overexpression leads to the changes relative to the control group. Compared with the normal control group (NC), the angiogenic capacity, including area, length, number of nodes, and number of branches of vessels, were all higher in the overexpression group than in the control group (*P* < 0.05) (Figure 5).

### Morpholino Knockdown of ACTA2 Causes Vascular Defects, Sprouting Angiogenesis, and Caudal Vein Plexus Formation Defects of Zebrafish

**FIGURE 6:**
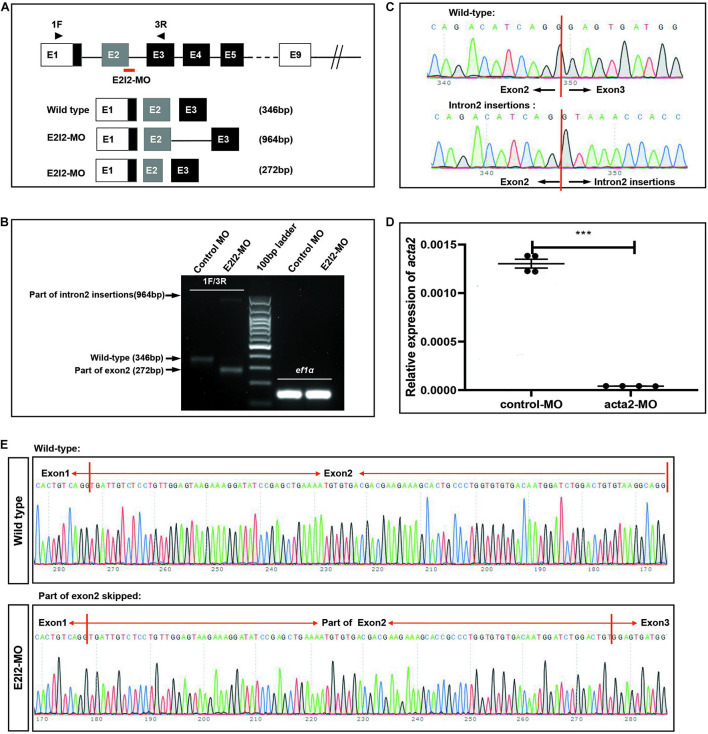
Effectiveness of acta2 knockdown was confirmed by RT-PCR and qRT-PCR. **(A)** The zebrafish acta2 gene was targeted by specific morpholino antisense to prevent proper splicing of exon 2 (E2I2-MO). **(B)** RT-PCR of acta2 transcript from control-MO and E2I2-MO morpholino-injected embryos 2 days after fertilization, demonstrating insertion of intron 2 and skipping of exon 2. **(C,E)** Sanger sequencing of both the wild type band and the intron 2-inserted band and the exon 2-skipped band validating the wild type sequence and the intron 2-inserted sequence **(C)** and the exon 2-skipped sequence **(E)**. **(D)** Quantitative measurements of acta2 expression levels measured by qRT-PCR (***P < 0.0001). MO-targeted down-regulation of acta2. dpf, days post-fertilization.

To investigate the vascular formation process affected by ACTA2, a zebrafish model with ACTA2 knockdown was constructed. The zebrafish acta2 gene was targeted by two specific morpholino antisense strategies to prevent either the translation of the zebrafish gene (ATG-MO) or proper splicing of exon2 (E2I2-MO). Primers 1F and 3R interrogate the presence of wild type (non-mutant) transcripts or those in which intron 2 has been inserted and exon 2 has been skipped (Figure 6A). And RT-PCR of acta2 transcript from control-MO and E2I2-MO morpholino-injected embryos 2 days after fertilization, demonstrating insertion of intron 2 and skipping of exon 2. Injection of 4 ng of acta2 morpholino alters the splicing between exon 2 and intron 2, as revealed by a shift in PCR bands between control and acta2 morpholino injected embryos (Figure 6B). As shown in Figures 6C,E, Sanger sequencing of both the wild type band and the intron 2-inserted band and the exon 2-skipped band validating the wild type sequence and the intron 2-inserted sequence and the exon 2-skipped sequence. Quantitative measurements of acta2 expression levels measured by quantitative qRT-PCR (Figure 6D). Samples were collected at 2-dpf after the introduction of 4ng of MO at the one-cell stage (*N* = 35).

**FIGURE 7:**
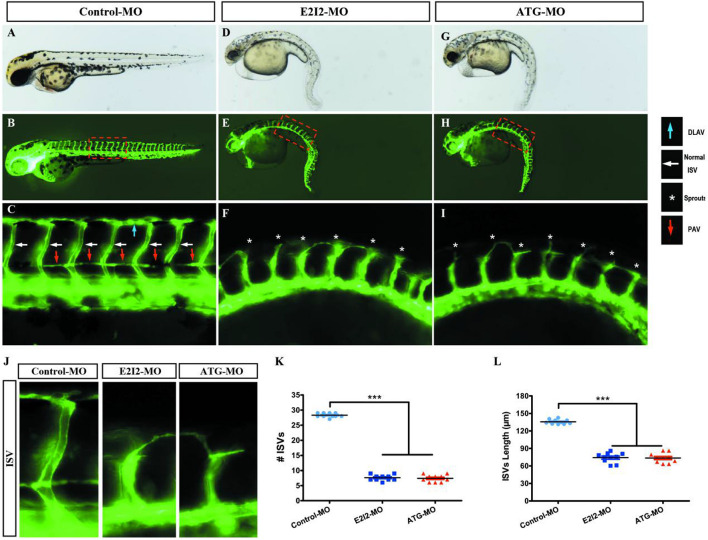
Morpholino knockdown of acta2 causes vascular defects. **(A–I)** Representative bright field and fluorescent images of Tg (fli1a:EGFP)y1 embryos at 50-hpf. **(B,C,E–H)** Image of trunk regions taken at 50-hpf, with the vascular structures visualized by eGFP fluorescence and labeled ISV (intersegmental vessel) and DLAV (dorsal longitudinal anastomotic vessel) showed regular development in the embryo injected with control MO. Compared with control MO, embryos injected with acta2-MO present a lower number of incomplete ISVs and ectopic sprouts (**F,I**, asterisk). In control embryos, the parachordal vessels (PAV) form normally (**C**, red arrows). Compared with control, MO knockdown acta2 prevents the parachordal vessels (PAV) formation, the precursor to the lymphatic system. The boxed regions are shown at higher magnification in the bottom panels. **(J–L)** Quantification of the number of complete ISVs or mean length of ISVs shows a significantly decrease in acta2 morphants. Columns, mean; SEM (*n* = 10; ANOVA) ***P < 0.0001. hpf, hours post-fertilization.

Image of trunk regions taken at 50-hpf, with the vascular structures visualized by eGFP fluorescence and labeled ISV (intersegmental vessel) and DLAV (dorsal longitudinal anastomotic vessel) showed regular development in the embryo injected with control MO (Figures 7A–C). Compared with control MO, embryos injected with acta2-MO present a lower number of incomplete ISVs and ectopic sprouts (Figure 7D–I). In control embryos, the parachordal vessels (PAV) form normally (C, red arrows). While compared with control, MO knockdown acta2 prevents the PAV formation, the precursor to the lymphatic system (Figures 7F,I). Quantification of the number of complete ISVs or mean length of ISVs shows a significantly decrease in acta2 morphants (Figures 7J–L).

**FIGURE 8:**
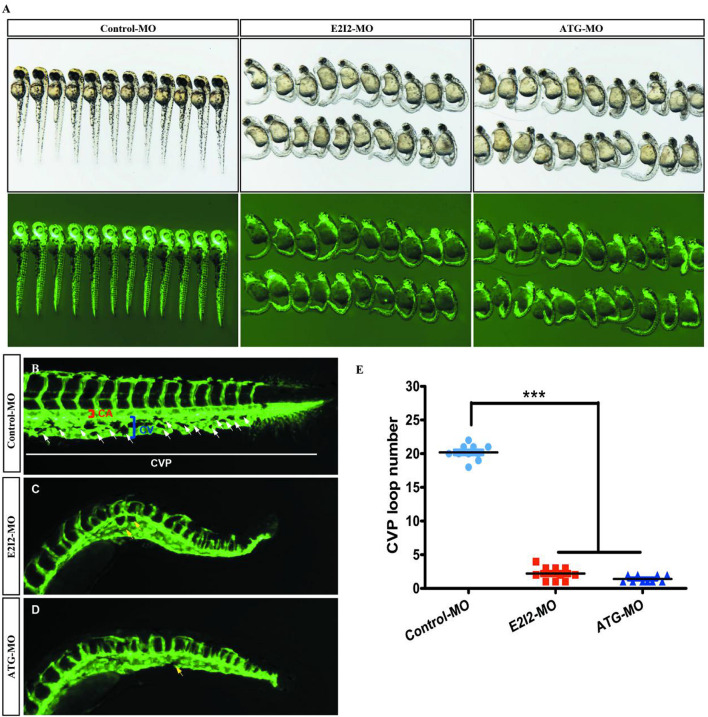
Acta2 knockdown impairs formation of the CVP in zebrafish. **(A)** In control embryos, caudal vein plexus (CVP) were formed honeycomb-like structures at the tail around 50 h post-fertilization (hpf). **(B–D)** Acta2 knockdown resulted in specific defects in CVP formation. **(E)** Quantification of loop number at CVP. Columns, mean; SEM (*n* = 10; ANOVA) *P < 0.05, **P < 0.01, ***P < 0.0001. CVP, caudal vein plexus; CA, caudal artery; CV, caudal vein.

Loss of acta2 impairs the formation of the CVP in zebrafish. In control embryos, caudal vein plexus (CVP) were formed honeycomb-like structures at the tail around 50 h post-fertilization (hpf) (Figures 8A,B). In contrast, acta2 knock down resulted in specific defects in CVP formation (Figures 8C,D).

According to these results, the direct effect of ACTA2 on early angiogenesis was identified *in vivo*. Morpholino knockdown of acta2 causes vascular defects, sprouting angiogenesis, and CVP (caudal vein plexus) formation defects of zebrafish (Figure 8E).

### Effect of ACTA2 on the Signaling Pathways in Zebrafish

**FIGURE 9:**
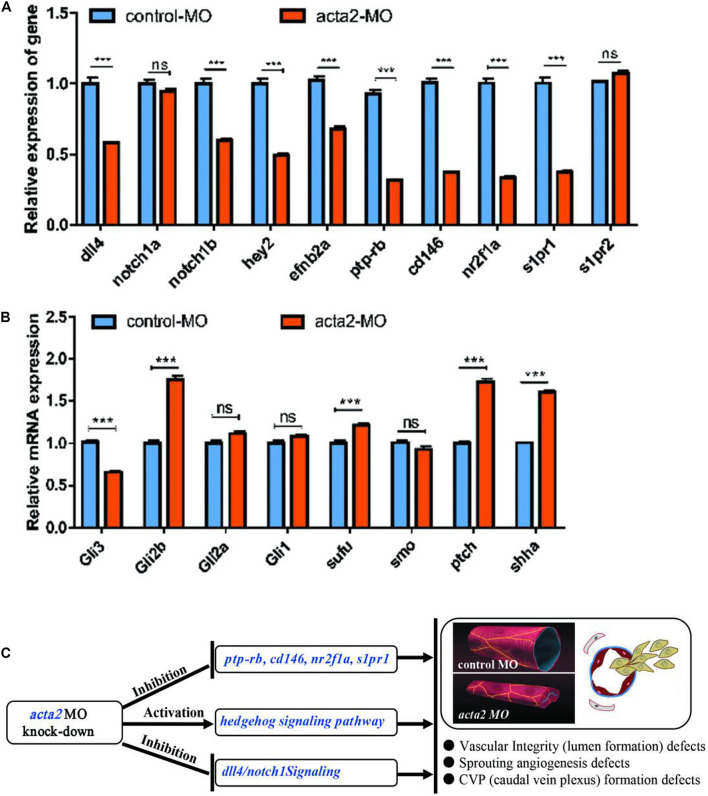
**(A)** Endogenous dll4, notch1a, notch1b, hey2, efnb2a, ptp-rb, cd146, nr2f1a, s1pr1 and s1pr2 in control and acta2 morphants assessed by qRT-PCR (*n* = 6–10 individual embryos). *** *P* < 0.001; ns, not significant. **(B)** Endogenous shha, ptch2, smo, Sufu, Gli1, Gli2a, Gli2b, and Gli3 in control and acta2 morphants assessed by qRT-PCR (*n* = 6–10 individual embryos). **(C)** Schematic model illustrating the MOA (mechanism of action) of acta2 in Zebrafish early development.

The knockdown of ACTA2 inhibited the Dll4/notch1signaling pathway, Ephrin-B2 signaling pathway, and vascular integrity-related molecules and activated the Hedgehog (Hh) signaling pathway. The expression of molecules related to each pathway in the control and ACTA2 knockdown groups was detected by qRT-PCR, revealing that dll4, notch1a, notch1b, hey2, efnb2a, ptp-rb, cd146, nuclear receptor subfamily 2 group F member 1a (nr2f1a), and s1pr1, which are associated with the Dll4/notch1signaling pathway, Ephrin-B2 signaling pathway, and vascular endothelial protein tyrosine phosphatase (VE-PTP), were all significantly downregulated (Figure 9A). The expression of shha, ptch2, Sufu, Gli1, and Gli2b, which are related to Hh signaling pathway were significantly increased (Figure 9B). PCR testing of the knockdown and control groups found that vascular malformation by ACTA2 knockdown may be due to the inhibition of the Dll4/notch1 signaling pathway, Ephrin-B2 signaling pathway, and vascular integrity-related molecules, as well as activation of the Hh signaling pathway.

This implies regulation of ACTA2 knockdown on the vascular malformation, via not only inhibited the Dll4/notch1 signaling pathway, Ephrin-B2 signaling pathway, and vascular integrity-related molecules but also upregulated the Hh signaling pathway. A schematic diagram was proposed to display the visualized relationship between expression levels and roles in the pathway (Figure 9C). However, the mechanism of crosstalk between cell signaling pathways *in vivo* is very complex, more work needs to be continued and discussed.

## Discussion

Venous malformations are the most common type of vascular malformation in clinical practice, accounting for approximately 66% of congenital vascular malformations, with a prevalence of approximately 1% and an incidence of 1/10,000 to 1/5000 (Dompmartin et al., 2010; Fowell et al., 2017). It may occur anywhere on the body, including the head and neck (40%), extremities (40%), and trunk (20%) (Dompmartin et al., 2010; Cahill and Nijs, 2011; Manoli et al., 2015). It is usually present from birth and progresses with growth and development (Judith et al., 2014; Lee et al., 2015). Its onset is not gendered specific (Carqueja et al., 2018), the exact etiology is unclear, and it was associated with mutated genes, such as TIE2 (TEK), PIK3CA, and MAP3K3 (Couto et al., 2015; Natynki et al., 2015; Castillo et al., 2016; Seront et al., 2018).

In recent years, the development of proteomics has brought about new ideas for the study of many diseases and provided a viable method to identify disease-specific protein markers. The functions of most genes depend on the proteins they encode, so the study of proteins in cells or organisms can help reveal the metabolic processes of cells and the life activities of the organism. Proteomics enables the quantification and analysis of hundreds to thousands of proteins in a single experiment, providing an important method to discover and validate biomarkers and discover new therapeutic targets on a large scale. Currently, mass spectrometry-based methods for proteomics quantification include TMT, isobaric tags for relative and absolute quantitation, multiple reaction monitoring, and parallel reaction monitoring, among which TMT is the most widely used (Wojdyla et al., 2015). Developed by Thermo Scientific, TMT is a relative and absolute quantitative technology based on *in vitro* isobaric isotope labeling. It has been widely used in proteomics research in recent years. The identification and quantification of proteins by tandem mass spectrometry has the advantages of antibody independence, simultaneous determination of multiple targets in multiple samples, and easy establishment of standard operation protocols (Zhang et al., 2016).

In this study, a total of 7408 peptides and 801 protein groups were identified by TMT technology, and 71 proteins with statistically significant differential expression were further selected, among which five were upregulated and 66 were downregulated. GO functional, KEGG pathway and structural domain annotation of the differentially expressed proteins revealed that their functional differences between the patients with venous malformations and controls primarily included biological regulation, cellular process, actin filament binding, molecular transducer activity, and binding and regulation of the actin cytoskeleton pathway, and the differences in CC were primarily focused on cytosol, organelles, cell part, and so on.

The HCS system generates a large number of images in a short period by fully automated high-speed microscopic imaging. Fully automated image analysis software extracts a large amount of data from these images, and the data management software is responsible for processing and analyzing these images and data to observe differences in cell morphology and biological functions (Anguissola et al., 2014). Celigo is a high-throughput screening system based on automatic image acquisition and image data analysis. The high throughput of the system is due to automatic target loading, high-speed imaging, and real-time automatic quantitative analysis. The software analyzes the images to determine the corresponding optical information of biological events, including coordinate position, signal intensity, and time information and their combinations and analyzes the biological changes, including cell morphology, cell movement, cell number, cell expression signal strength, cell cycle, apoptosis, and other common biological phenomena. In this study, five highly and 15 lowly expressed proteins were selected based on bioinformatic analysis and proteomics results and were included in HCS for the RNAi functional screening test. This study identified ACTA2 as the most differentially expressed gene associated with vascular endothelial cell proliferation, which was further investigated by tissue expression, cellular function assays, and *in vivo* studies.

The ACTA2 gene is located at chromosome 10q22-q24. Its primary function is to encode actin and it is widely expressed in most cells. It encodes ACTA2, actin with multiple aliases, including alpha-actin, alpha-actin-2, aortic smooth muscle, and alpha-smooth muscle actin. Mutations in this gene have been found to cause a variety of vascular diseases, such as thoracic aortic disease, coronary artery disease, stroke, moyamoya disease, and multisystem smooth muscle dysfunction syndrome (Guo et al., 2009). ACTA2 protein is a ubiquitous cytoskeletal protein that is involved in cell activation, differentiation, and migration. It constitutes a cellular scaffold, maintains cell shape, and mediates intracellular signaling and protein synthesis (Xu et al., 2019). Previous studies have shown that aberrant expression of ACTA2 promotes the invasion and migration of eutopic endometrial stromal cells (Rockey et al., 2013) and leads to significantly enhanced metastasis in lung cancer (Lee et al., 2013). The deletion of ACTA2 leads to reduced cell motility and contraction of myofibroblasts during cranial injury healing (Zhang et al., 2020). In cardiovascular diseases, defects in the function of actin or myosin (ACTA2 or MYH11) result in diminished actin–myosin interactions and lead to diseases (Fang et al., 2017). In this study, the deletion of ACTA2 resulted in the disruption of HMEC proliferation, leading to a lack of vascular development and causing venous malformations. We found that overexpression of ACTA2 improved proliferation, migration, invasion, and angiogenesis of vascular endothelial cells, and their abnormal proliferation was inhibited.

In the field of vascular development research, as an emerging animal model, zebrafish have unique molecular and histological advantages due to easy manipulation, rapid growth and development, easy observation of blood vessels, and high genetic homology. The expression and distribution of relevant vascular endothelial markers, early vasculogenesis, and maturation can be directly and dynamically observed by transgenic fluorescent labeling techniques (Westerfield, 2007), in which the embryonic caudal vein plexus (CVP) is considered one of the representative models for simulating early vein development. CVP is the basis of the mature caudal vein. At the early stage of development, it is shown as a large aggregation of endothelial cell clusters, followed by an initial sinus lumen morphology 24 h after embryonic fertilization. Endothelial cells proliferate actively outside the CVP and extend to the pseudopodia. At the same time, the cavities of the sinuses are gradually reconstructed, eventually forming a mature caudal vein lumen (Wakayama et al., 2015). The vascular development of this process can be visually recorded based on the fluorescence technique of visualization and the transparent nature of the fish body. In this study, we constructed a zebrafish model with ACTA2 knockdown and observed that ACTA2 knockdown caused defects in vascular integrity, neovascularization, and CVP formation in zebrafish. This indicates that ACTA2 deletion severely affects vascular integrity and causes abnormal vein development.

Notch signaling is an evolutionary conserved, intercellular signaling mechanism that plays myriad roles during vascular development and physiology in vertebrates. Defects in Notch signaling also cause inherited vascular diseases (Gridley, 2010). Delta-like ligand 4 (DLL4) is the only Notch1 ligand specifically present in vascular endothelial cells that regulate vascular sprouting and branching morphology (Hu et al., 2014). Notch1 is the most studied receptor in the Notch signaling pathway, which regulates the biological behavior of cells through interactions between neighboring cells. Aberrant activation of Notch 1 may lead to excessive cell proliferation and invasion (Mckeage et al., 2019). The expression of key molecules, such as DLL4 and Notch1b, was significantly reduced in zebrafish in the ACTA2 knockdown group in this study, suggesting that ACTA2 deletion may lead to the inhibition of the DLL4-Notch1 signaling pathway, resulting in defective vascular development.

Ephrin-B2 is a well-recognized axon guidance factor that affects angiogenesis primarily by regulating endothelial cell function. It plays a crucial role in vascular development and revascularization during embryonic and adult life (Birgit et al., 2010). The Ephrin-B2 signaling pathway can cooperate with VEGF to coregulate vascular growth. Selective inhibition of the Ephrin-B2 signaling pathway can lead to a significant reduction in sprouting angiogenesis (Wang Y. et al., 2010). In this study, the expression of Ephrin-B2 signaling pathway-related molecules was significantly reduced in zebrafish vascular tissues of the ACTA2 knockdown group, indicating that ACTA2 deletion also affects the inhibition of this pathway, causing abnormal angiogenic function.

Vascular integrity is the basis of vascular endothelial cell function and is closely associated with the development of numerous vascular diseases (Dejana et al., 2009). VE-PTP, nr2f1a, and Sphingosine 1-phosphate receptor are closely associated with vascular integrity (Wang C. et al., 2010; Hayashi et al., 2013; Mendelson et al., 2013; Wu et al., 2014). In this study, the expression of molecules related to vascular integrity was significantly reduced in the vascular tissue of zebrafish in the ACTA2 knockdown group, implying that ACTA2 deletion led to the disruption of vascular integrity.

The Hh signaling pathway is essential for embryonic development and plays a key role in human tissue maintenance, renewal, and regeneration. An abnormally activated Hh signaling pathway is associated with the development of several tumors and other diseases (Xin et al., 2018). The expression levels of important transcriptional factors and ligands in this pathway, including shha, ptch2, Sufu, and Gli2b, were significantly increased in the ACTA2 knockdown group of zebrafish, indicating that ACTA2 deletion caused abnormal activation of this pathway and led to abnormal vascular development.

In summary, this study screened differentially expressed proteins in venous malformation and normal human tissues by proteomics and identified the most significantly differentially expressed gene, ACTA2, by HCS. Compared with healthy people, the expression of ACTA2 was significantly reduced in patients with venous malformations. We also established a cell model to overexpress ACTA2 in HMEC-1 and revealed that the abnormalities of cell proliferation, metastasis, invasion, and angiogenesis were reversed by ACTA2 overexpression. We also established a zebrafish animal model and performed *in vivo* experiments, resulting in the finding that knockdown of ACTA2 in zebrafish results in defective vascular development, disrupted vascular integrity, and abnormal development of the caudal vein. PCR testing of the knockdown and control groups found that vascular malformation by ACTA2 knockdown may be due to the inhibition of the Dll4/notch1 signaling pathway, Ephrin-B2 signaling pathway, and vascular integrity-related molecules, as well as activation of the Hh signaling pathway. ACTA2 may be a potential target for the diagnosis and treatment of disseminated venous malformations. However, the mechanism of crosstalk between cell signaling pathways *in vivo* is very complex, more work needs to be continued and discussed.

## Data Availability Statement

The datasets presented in this study can be found in online repositories. The data presented in the study are deposited in the ProteomeXchange repository, accession number PXD028848.

## Ethics Statement

The studies involving human participants were reviewed and approved by Shandong Provincial Ethics Committee. Written informed consent to participate in this study was provided by the participants’ legal guardian/next of kin. The animal study was reviewed and approved by Shandong Provincial Hospital Ethics Committee. Written informed consent was obtained from the owners for the participation of their animals in this study.

## Author Contributions

RH and JB conceived and designed the experiments. SW analyzed the experimental results. RL, GX, and JZ collected and analyzed the data. ZZ, JL, YW, and HL performed the experiments. SW wrote the manuscript. All authors have given approval to the final version of the manuscript.

## Conflict of Interest

The authors declare that the research was conducted in the absence of any commercial or financial relationships that could be construed as a potential conflict of interest.

## Publisher’s Note

All claims expressed in this article are solely those of the authors and do not necessarily represent those of their affiliated organizations, or those of the publisher, the editors and the reviewers. Any product that may be evaluated in this article, or claim that may be made by its manufacturer, is not guaranteed or endorsed by the publisher.

## Footnotes

1
http://www.gene-tools.com/

2
http://rsbweb.nih.gov/ij/

## Abbreviations

VM: venous malformations
HCS: high connotation functional gene screening
TMT: Tandem Mass Tag
HMEC: human microvascular endothelial cell
GO: Gene Ontology
GFP: green fluorescent protein
MO: morpholino
CVP: caudal vein plexus
PAV: parachordal vessels
ISV: intersegmental vessel
DLAV: dorsal longitudinal anastomotic vessel.
